# Tuberculosis — United States, 2022

**DOI:** 10.15585/mmwr.mm7212a1

**Published:** 2023-03-24

**Authors:** Kimberly R. Schildknecht, Robert H. Pratt, Pei-Jean I. Feng, Sandy F. Price, Julie L. Self

**Affiliations:** ^1^Epidemic Intelligence Service, CDC; ^2^Division of Tuberculosis Elimination, National Center for HIV, Viral Hepatitis, STD, and TB Prevention, CDC.

Incidence of reported tuberculosis (TB) decreased gradually in the United States during 1993–2019, reaching 2.7 cases per 100,000 persons in 2019. Incidence substantially declined in 2020 to 2.2, coinciding with the COVID-19 pandemic (*1*). Proposed explanations for the decline include delayed or missed TB diagnoses, changes in migration and travel, and mortality among persons susceptible to TB reactivation (*1*). Disparities (e.g., by race and ethnicity) in TB incidence have been described (*2*). During 2021, TB incidence partially rebounded (to 2.4) but remained substantially below that during prepandemic years, raising concerns about ongoing delayed diagnoses (*1*). During 2022, the 50 U.S. states and the District of Columbia (DC) provisionally reported 8,300 TB cases to the National Tuberculosis Surveillance System. TB incidence was calculated using midyear population estimates and stratified by birth origin and by race and ethnicity. During 2022, TB incidence increased slightly to 2.5 although it remained lower than during prepandemic years.* Compared with that in 2021, TB epidemiology in 2022 was characterized by more cases among non–U.S.-born persons newly arrived in the United States; higher TB incidence among non-Hispanic American Indian or Alaska Native (AI/AN) and non-Hispanic Native Hawaiian or other Pacific Islander (NH/OPI) persons and persons aged ≤4 and 15–24 years; and slightly lower incidence among persons aged ≥65 years. TB incidence appears to be returning to prepandemic levels. TB disparities persist; addressing these disparities requires timely TB diagnosis and treatment to interrupt transmission and prevention of TB through treatment of latent TB infection (LTBI).

Health departments in the 50 U.S. states and DC electronically report verified TB cases to CDC based on the Council of State and Territorial Epidemiologists’ surveillance case definition.^†^ Midyear U.S. Census Bureau population estimates^§^ are used to calculate national, state-level, and age-stratified TB incidence. Persons with TB are grouped by self-reported race and ethnicity according to federal guidelines.^¶^ Persons reporting Hispanic ethnicity are categorized as Hispanic or Latino (Hispanic) irrespective of race. Non-Hispanic persons are categorized by race; non-Hispanic persons who reported more than one race are categorized as “multiple race.” Midyear population estimates from the Current Population Survey** are used to calculate incidence by U.S. birth origin (U.S.-born versus non–U.S.-born)^††^ and by race and ethnicity. This activity was reviewed by CDC and was conducted consistent with applicable federal law and CDC policy.^§§^

During 2022, 8,300 TB cases were reported in the United States, compared with 7,874 during 2021. TB incidence during 2022 increased slightly to 2.5 per 100,000 persons, compared with 2.4 during 2021. Consistent with previous years (*1*), in 2022, California reported the highest number of TB cases (1,843) and Alaska reported the highest TB incidence (13.1) (Table 1).

**TABLE 1:** Number of tuberculosis disease cases and tuberculosis incidence, by jurisdiction — National Tuberculosis Surveillance System, United States, 2019–2022

Jurisdiction	No. of cases*				Incidence^†^			
	2019	2020	2021	2022	2019	2020	2021	2022
Alabama	87	72	91	66	1.8	1.4	1.8	1.3
Alaska	58	58	58	96	7.9	7.9	7.9	13.1
Arizona	183	136	129	154	2.5	1.9	1.8	2.1
Arkansas	64	59	69	69	2.1	2.0	2.3	2.3
California	2,110	1,703	1,749	1,843	5.4	4.3	4.5	4.7
Colorado	66	52	58	57	1.1	0.9	1.0	1.0
Connecticut	67	54	54	67	1.9	1.5	1.5	1.8
Delaware	19	17	41	13	1.9	1.7	4.1	1.3
District of Columbia	24	19	18	15	3.4	2.8	2.7	2.2
Florida	558	412	500	536	2.6	1.9	2.3	2.4
Georgia	302	220	222	256	2.8	2.1	2.1	2.3
Hawaii	99	92	106	101	7.0	6.3	7.3	7.0
Idaho	7	8	5	11	0.4	0.4	0.3	0.6
Illinois	326	216	254	298	2.6	1.7	2.0	2.4
Indiana	108	92	127	99	1.6	1.4	1.9	1.4
Iowa	52	39	49	60	1.6	1.2	1.5	1.9
Kansas	37	37	43	52	1.3	1.3	1.5	1.8
Kentucky	65	66	57	70	1.5	1.5	1.3	1.6
Louisiana	88	99	86	89	1.9	2.1	1.9	1.9
Maine	18	17	14	20	1.3	1.2	1.0	1.4
Maryland	209	149	197	152	3.5	2.4	3.2	2.5
Massachusetts	178	142	151	153	2.6	2.0	2.2	2.2
Michigan	131	101	137	120	1.3	1.0	1.4	1.2
Minnesota	148	117	134	132	2.6	2.0	2.3	2.3
Mississippi	57	43	46	54	1.9	1.5	1.6	1.8
Missouri	70	79	77	69	1.1	1.3	1.2	1.1
Montana	2	4	3	6	0.2	0.4	0.3	0.5
Nebraska	17	33	22	28	0.9	1.7	1.1	1.4
Nevada	53	57	61	62	1.7	1.8	1.9	2.0
New Hampshire	6	12	12	11	0.4	0.9	0.9	0.8
New Jersey	309	245	289	286	3.5	2.6	3.1	3.1
New Mexico	41	29	24	30	2.0	1.4	1.1	1.4
New York	746	605	683	714	3.8	3.0	3.4	3.6
North Carolina	185	159	178	163	1.8	1.5	1.7	1.5
North Dakota	18	10	15	5	2.4	1.3	1.9	0.6
Ohio	149	132	151	148	1.3	1.1	1.3	1.3
Oklahoma	73	67	69	80	1.8	1.7	1.7	2.0
Oregon	70	67	79	70	1.7	1.6	1.9	1.7
Pennsylvania	198	157	166	173	1.5	1.2	1.3	1.3
Rhode Island	14	7	17	17	1.3	0.6	1.5	1.6
South Carolina	80	67	87	101	1.6	1.3	1.7	1.9
South Dakota	16	16	12	10	1.8	1.8	1.3	1.1
Tennessee	129	113	84	107	1.9	1.6	1.2	1.5
Texas	1,154	879	996	1,089	4.0	3.0	3.4	3.6
Utah	27	29	17	33	0.8	0.9	0.5	1.0
Vermont	4	3	3	3	0.6	0.5	0.5	0.5
Virginia	191	169	160	195	2.2	2.0	1.8	2.2
Washington	221	163	199	253	2.9	2.1	2.6	3.2
West Virginia	9	13	6	11	0.5	0.7	0.3	0.6
Wisconsin	51	35	66	52	0.9	0.6	1.1	0.9
Wyoming	1	0	3	1	0.2	0	0.5	0.2
**Total**	**8,895**	**7,170**	**7,874**	**8,300**	**2.7**	**2.2**	**2.4**	**2.5**

* Case counts are based on data reported to the National Tuberculosis Surveillance System as of March 6, 2023.

^†^ Incidence is calculated as cases per 100,000 persons using midyear population estimates from the U.S. Census Bureau. Short-term projections from the monthly population estimates by age, sex, race, and Hispanic origin were used for 2022 population, 2021 vintage population estimates were used for 2021 and 2020, and 2010 vintage population estimates were used for 2019. https://www.census.gov/programs-surveys/popest/data/tables.html; https://www.census.gov/programs-surveys/popest/data/tables.2019.List_58029271.html#list-tab-List_58029271

In 2022, 73% (6,009 of 8,248 TB cases in persons for whom birth origin was known) of TB cases occurred among non–U.S.-born persons,^¶¶^ compared with 72% in 2021. Among U.S.-born persons, TB incidence was 0.8 during both 2021 and 2022; among non–U.S.-born persons, incidence increased slightly from 12.6 in 2021 to 12.8 in 2022 (Figure) (Table 2). Among 2,239 U.S.-born persons with TB in 2022, 673 (30%) identified as non-Hispanic Black or African American (Black), 578 (26%) as Hispanic, 568 (25%) as non-Hispanic White (White), 182 (8%) as non-Hispanic Asian (Asian), 110 (5%) as AI/AN, and 52 (2%) as NH/OPI; 76 (3%) identified as multiple race or had unknown race and ethnicity. Among these groups, incidence was highest among NH/OPI persons (6.6), followed by AI/AN (4.4), Asian (2.2), and Black persons (1.9) and was lowest among White persons (0.3). Compared with that in 2021, incidence in 2022 increased 63% among Asian persons, 26% among NH/OPI persons, 16% among AI/AN persons, and 7% among Hispanic persons. Incidence declined 9% among Black persons, and 10% among White persons.***

**FIGURE:**
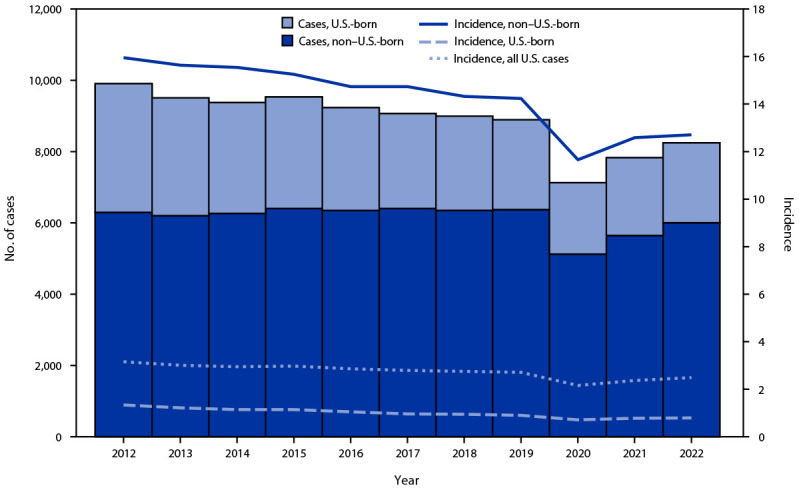
Tuberculosis disease cases* and incidence,^†^ by patient U.S. birth origin status^§^^,^^¶^ — National Tuberculosis Surveillance System, United States, 2012–2022 * Case counts are based on data from the National Tuberculosis Surveillance System as of March 6, 2023. ^†^ Cases per 100,000 persons. The Current Population Survey provides the population denominators used to calculate tuberculosis incidence according to national origin and racial and ethnic group. https://www.census.gov/programs-surveys/cps.html (Accessed February 3, 2023). ^§^ A person is considered U.S.-born if eligible for U.S. citizenship at birth, regardless of place of birth. Birth origin was missing or unknown for 232 (2.8%) cases during 2022. Among those, 180 (77.6%) had country of birth reported, and birth origin was defined as U.S.-born for persons reporting birth in the United States or U.S. territories and as non–U.S.-born for persons born outside the United States and its territories. ^¶^ Persons for whom birth origin was unknown (range = 2 [2012] to 52 [2022]) were excluded.

**TABLE 2:** Demographic and risk characteristics of persons with tuberculosis and number and incidence of tuberculosis cases — National Tuberculosis Surveillance System, United States, 2021–2022

Characteristic	No. of TB cases* (%)^†^			TB incidence^§^		
	2021	2022	% Change 2021 to 2022	2021	2022	% Change 2021 to 2022
**Total**	**7,874**	**8,300**	**5.4**	**2.4**	**2.5**	**5.0**
**Age group, yrs**						
≤4	160 (2.0)	202 (2.4)	26.3	0.8	1.1	28.8
5–14	156 (2.0)	161 (1.9)	3.2	0.4	0.4	4.4
15–24	676 (8.6)	840 (10.1)	24.3	1.6	1.9	23.7
25–44	2,265 (28.8)	2,431 (29.3)	7.3	2.5	2.7	7.1
45–64	2,409 (30.6)	2,419 (29.2)	0.4	2.9	2.9	1.1
≥65	2,208 (28.0)	2,244 (27.0)	1.6	4.0	3.9	−1.8
**Birth origin,**^¶^ **race and ethnicity**						
**U.S.-born**						
AI/AN, non-Hispanic	86 (3.9)	110 (4.9)	27.9	3.8	4.4	15.8
Asian, non-Hispanic	112 (5.1)	182 (8.1)	62.5	1.4	2.2	63.2
Black or African American, non-Hispanic	743 (34.1)	673 (30.1)	−9.4	2.0	1.9	−9.1
NH/OPI, non-Hispanic	40 (1.8)	52 (2.3)	30.0	5.2	6.6	26.2
White, non-Hispanic	634 (29.1)	568 (25.4)	−10.4	0.3	0.3	−10.4
Hispanic or Latino	539 (24.7)	578 (25.8)	7.2	1.3	1.4	6.6
Unknown race and ethnicity or multiple races	28 (1.3)	76 (3.4)	NA	NA	NA	NA
**Subtotal**	**2,182 (100.0)**	**2,239 (100.0)**	**2.6**	**0.8**	**0.8**	**2.4**
**Non–U.S.-born**						
AI/AN, non-Hispanic	1 (0)	2 (0)	100.0	1.3	4.3	221.3
Asian, non-Hispanic	2,709 (47.8)	2,632 (43.8)	−2.8	23.8	22.0	−7.5
Black or African American, non-Hispanic	674 (11.9)	625 (10.4)	−7.3	15.5	13.7	−11.6
NH/OPI, non-Hispanic	75 (1.3)	103 (1.7)	37.3	23.3	27.8	19.5
White, non-Hispanic	249 (4.4)	276 (4.6)	10.8	3.2	3.4	7.4
Hispanic or Latino	1,847 (32.6)	2,194 (36.5)	18.8	8.9	10.1	12.6
Unknown race and ethnicity or multiple races	109 (1.9)	177 (2.9)	NA	NA	NA	NA
**Subtotal**	**5,664 (100.0)**	**6,009 (100.0)**	**6.1**	**12.6**	**12.8**	**1.1**
Unknown birth origin**	28 (0.4)	52 (0.6)	NA	NA	NA	NA
**Interval from initial U.S. arrival to TB diagnosis, yrs**						
<1	553 (9.8)	992 (16.5)	79.4	NA	NA	NA
1–10	1,642 (29.0)	1,528 (25.4)	−6.9	NA	NA	NA
>10	2,845 (50.2)	2,821 (46.9)	−0.8	NA	NA	NA
Unknown	624 (11.0)	668 (11.1)	7.1	NA	NA	NA
**HIV-positive at time of diagnosis**	302 (4.3)	327 (4.7)	8.3	NA	NA	NA
**Experienced homelessness during previous year**	352 (4.5)	380 (4.8)	8.0	NA	NA	NA
**Correctional facility resident at diagnosis**	178 (2.3)	286 (3.5)	60.7	NA	NA	NA
**Long-term care facility resident at diagnosis**	109 (1.4)	139 (1.7)	27.5	NA	NA	NA

**Abbreviations**: AI/AN = American Indian or Alaska Native; NA = not applicable; NH/OPI = Native Hawaiian or other Pacific Islander; TB = tuberculosis.

* Case counts are based on data reported to the National Tuberculosis Surveillance System as of March 6, 2023.

^†^ Percentages are calculated only among patients with available data, except for the years after U.S. arrival. Age was missing or unknown for zero cases in 2021 and three cases in 2022; origin of birth remained missing or unknown for 28 cases in 2021 and 52 cases in 2022; race and ethnicity was missing or unknown for 56 cases in 2021 and 175 cases in 2022; HIV testing results were missing or unknown for 842 cases in 2021 and 1,270 cases in 2022; whether a person was experiencing homelessness was missing or unknown for 60 cases in 2021 and 301 cases in 2022; whether a person was residing in a correctional facility was missing or unknown for 92 cases in 2021 and 215 cases in 2022; and whether a person was residing in a long-term care facility was missing or unknown for 81 cases in 2021 and 215 cases in 2022.

^§^ Incidence is calculated as cases per 100,000 persons using midyear population estimates from the U.S. Census Bureau. Short-term projections from the monthly population estimates by age, sex, race, and Hispanic origin were used for 2022 population, 2021 vintage population estimates were used for 2021 and 2020, and 2010 vintage population estimates were used for 2019. https://www.census.gov/programs-surveys/popest/data/tables.html; https://www.census.gov/programs-surveys/popest/data/tables.2019.List_58029271.html#list-tab-List_58029271

^¶^ A person is considered U.S.-born if eligible for U.S. citizenship at birth, regardless of place of birth. Birth origin was missing or unknown for 232 (2.8%) cases during 2022. Among those, 180 (77.6%) had country of birth reported, and birth origin was defined as U.S.-born for persons reporting birth in the United States or U.S. territories and as non–U.S.-born for persons born outside the United States and its territories.

** Excluded from race and ethnicity subtotals.

In 2022, 6,009 TB cases occurred among non–U.S.-born persons; >80% of these cases were among Asian (2,632; 44%) or Hispanic (2,194; 37%) persons. The remaining cases occurred among Black (625; 10%), White (276; 5%), and NH/OPI (103; 2%) persons, and multiple race persons or persons whose race and ethnicity were unknown (177; 3%). In 2022, similar to that among U.S.-born persons, the highest TB incidence among non–U.S.-born persons (27.8) was among NH/OPI persons. The next highest incidence (22.0) occurred among Asian persons, followed by Black (13.7), Hispanic (10.1), AI/AN (4.3) and White (3.4) persons. Among these groups, the largest increase in incidence from 2021 to 2022 (221%) occurred among AI/AN persons, followed by NH/OPI (20%), Hispanic (13%), and White (7%) persons. Incidence declined 12% among Black persons and 7% among Asian persons in 2022.

Among non–U.S.-born persons with TB in 2022, 16.5% (992) received a diagnosis <1 year after their initial arrival in the United States, compared with 9.8% (553) during 2021. A slightly lower number and percentage of persons with newly diagnosed TB were living in the United States for >10 years in 2022 (2,821; 46.9%) compared with 2021 (2,845; 50.2%). 

By age group, TB incidence in 2022 was highest among persons aged ≥65 years (3.9), followed by persons aged 45–64 (2.9), 25–44 (2.7), 15–24 (1.9), ≤4 (1.1), and 5–14 years (0.4). Compared with 2021, 2022 had the largest increase in incidence among persons aged ≤4 (28.8%) and 15–24 years (23.7%); persons aged ≥65 years were the only group that experienced a decrease (1.8%). Among 84.7% of persons with TB that had a known HIV status, 4.7% were coinfected in 2022 compared with 4.3% in 2021. Among persons with TB, increased percentages reported experiencing homelessness within 12 months preceding diagnosis (4.8%) and residing in a correctional facility (3.5%) or long-term care facility (1.7%) at the time of diagnosis in 2022, compared with 2021 (Table 2).^†††^

## Discussion

U.S. TB incidence increased during 2022, compared with that during 2020 and 2021, but remained lower than incidence during the prepandemic years; after a substantial 20.2% decline in 2020 and partial rebound (9.8% increase) in 2021 (*1*), incidence appears to be returning to prepandemic levels among U.S.-born and non–U.S.-born populations.

COVID-19–associated mortality was high among persons aged ≥65 years, which might account, in part, for the lower TB incidence observed among that population (*3*). Even though the decrease in TB incidence was small, reduction of the population aged ≥65 years at risk for TB might have similar effects on TB incidence in future years. The increase in TB incidence among children aged ≤4 years might represent both recent transmission in the United States and infection in countries with higher TB incidence. An analysis of TB incidence among indigenous persons during 2009–2019 found a higher prevalence of underlying chronic medical conditions, and TB incidence was at least 10 times higher among AI/AN and NH/OPI persons than among White persons (*2*). These factors likely contributed to the higher TB incidence in these populations in this report. Among non–U.S.-born persons with TB, the higher proportion reported <1 year after arrival in the United States might reflect greater migration from higher TB incidence areas than what existed at the beginning of the pandemic.^§§§^

Although preventing TB transmission in the United States remains a priority, >80% of U.S. TB cases are attributed to reactivation of LTBI (*1*). To achieve TB elimination in the United States, the U.S. Preventive Services Task Force recommends testing and treatment among populations at higher risk for LTBI, including non–U.S.-born persons and persons in congregate living settings (*4*). To treat LTBI, CDC recommends short-course (3- or 4-month), rifamycin-based regimens (*5*). Shorter regimens are also available to treat TB: in 2022, CDC recommended a 4-month treatment regimen for drug-susceptible pulmonary TB as an alternative to the standard 6-month regimen (*6*). Shorter treatment durations improve treatment adherence and completion (*5**,**6*).

Higher TB incidence among AI/AN and NH/OPI persons represents an ongoing health disparity (*2*) in the United States. Alaska reported an increase of TB in 2022 and identified Alaska Native persons as among those at highest risk for TB (*7*). CDC is working to raise awareness about TB and LTBI among communities at risk for TB and their health care providers through the Think. Test. Treat TB campaign,^¶¶¶^ which offers resources in multiple languages for general audiences and health care providers.**** CDC also partners with community health clinics and organizations, including the TB Elimination Alliance,^††††^ to address TB health disparities through education and innovation.

Higher proportions of TB cases among persons experiencing homelessness or residing in correctional or long-term care facilities might be partially explained by transmission events in congregate settings. For example, gaps in TB infection control practices when resources were diverted to COVID-19 prevention and control efforts likely led to a TB outbreak in at least one state’s prison system during 2021–2022 (*8*).

The findings in this report are subject to at least two limitations. First, this analysis and case counts are based on provisional 2022 TB surveillance data and might change. Second, rates are calculated with population estimates that are subject to future refinement.

Knowledge of the effects of the COVID-19 pandemic on U.S. TB epidemiology is evolving. As COVID-19 incidence declines, TB remains an important public health challenge characterized by persistent inequities, particularly among AI/AN and NH/OPI populations, persons experiencing homelessness, and persons who are incarcerated. Timely detection and treatment of TB and LTBI among persons at risk are needed to achieve TB elimination in the United States.

**Summary**

**What is already known about this topic?**

During the early COVID-19 pandemic (2020), U.S. incidence of reported tuberculosis (TB) substantially declined. Incidence partially rebounded in 2021 but remained lower than incidence during prepandemic years.

**What is added by this report?**

During 2022, reported TB incidence increased slightly. Among non–U.S.-born persons with TB, the proportion who had recently arrived in the United States increased. Higher TB incidence among American Indian or Alaska Native and Native Hawaiian or other Pacific Islander persons compared with other race and ethnicity groups represents an ongoing health disparity.

**What are the implications for public health practice?**

TB incidence is returning to prepandemic levels. TB diagnosis and treatment to interrupt transmission and prevention of TB through treatment of latent TB infection are critical to U.S. TB elimination efforts.

## Footnotes

* This report is limited to National Tuberculosis Surveillance System data provisionally reported by the 50 U.S. states and DC as of March 6, 2023. Updated data will be available in CDC’s annual TB surveillance report later in 2023.

† TB cases are verified based on the case definition for public health surveillance, which includes laboratory criteria, clinical criteria, or provider diagnosis. https://ndc.services.cdc.gov/case-definitions/tuberculosis-2009/

§ Short-term projections from the monthly population estimates by age, sex, race, and Hispanic origin were used for 2022 population, 2021 vintage population estimates were used for 2021 and 2020, and 2010 vintage population estimates were used for 2012–2019. https://www.census.gov/programs-surveys/popest/data/tables.html; https://www.census.gov/programs-surveys/popest/data/tables.2019.List_58029271.html#list-tab-List_58029271

¶
https://www.census.gov/topics/population/race/about.html

**
https://www.census.gov/programs-surveys/cps.html

†† A person is considered U.S.-born if eligible for U.S. citizenship at birth, regardless of place of birth. Birth origin was missing or unknown for 232 (2.8%) cases during 2022. Among those, 180 (77.6%) had country of birth reported, and birth origin was defined as U.S.-born for persons reporting birth in the United States or U.S. territories and as non–U.S.-born for persons born outside the United States and its territories.

§§ 45 C.F.R. part 46.102(l)(2), 21 C.F.R. part 56; 42 U.S.C. Sect. 241(d); 5 U.S.C. Sect. 552a; 44 U.S.C. Sect. 3501 et seq.

¶¶ Proportions using birth origin are calculated excluding those with missing data.

*** Percentage change is calculated from unrounded numbers. For demographic groups with small populations (e.g., non–U.S.-born AI/AN persons), changes in incidence rates should be interpreted cautiously because of the increased volatility of these rates.

††† Percentages are calculated using cases with complete data for each of these three individual variables.

§§§
https://www.census.gov/library/stories/2022/12/net-international-migration-returns-to-pre-pandemic-levels.html

¶¶¶
https://www.cdc.gov/thinktesttreattb

****
https://www.cdc.gov/thinktesttreattb/resources.html

††††
https://tbeliminationalliance.org/
